# Basal ganglia lesions in subacute sclerosing panencephalitis

**DOI:** 10.1590/S1980-57642012DN06040014

**Published:** 2012

**Authors:** Kelson James Almeida, Sonia Maria Dozzi Brucki, Maria Irma Seixas Duarte, Carlos Augusto Gonçalves Pasqualucci, Sérgio Rosemberg, Ricardo Nitrini

**Affiliations:** 1 MD, Behavioral and Cognitive Neurology Unit and Cognitive Disorders Reference Center (CEREDIC), Department of Neurology, São Paulo SP, Brazil;; 2 Laboratory of Transmissible Diseases Pathology, Department of Pathology, São Paulo SP, Brazil;; 3 Department of Pathology, School of Medicine of the University of São Paulo (FMUSP) São Paulo SP, Brazil.

**Keywords:** subacute sclerosing panencephalitis, measles, magnetic resonance imaging

## Abstract

The parieto-occipital region of the brain is the most frequently and severely affected in subacute sclerosing panencephalitis (SSPE). The basal ganglia, cerebellum and corpus callosum are less commonly involved. We describe a patient with SSPE confirmed by neuropathology based on brain magnetic resonance imaging showing extensive basal ganglia involvement and no significant involvement of other cortical structures. Though rarely described in SSPE, clinicians should be aware of this involvement. SSPE should be kept in mind when changes in basal ganglia signal are seen on brain magnetic resonance imaging with or without involvement of other regions of the human brain to avoid erroneous etiological diagnosis of other pathologies causing rapidly progressive dementia.

## Abstract (pt)

A região parietooccipital é mais frequente e gravemente acometida na panencefalite esclerosante subaguda (PEESA). Os gânglios da base, cerebelo e corpo caloso são menos envolvidos. Descrevemos um paciente com PEESA confirmada por neuropatologia com imagens de ressonância magnética (RNM) evidenciando acometimento extenso dos gânglios da base sem envolvimento de outras estruturas corticais. Embora raramente descritas nesta doença, deve-se ficar atento para tal acometimento e PEESA deve ser lembrada quando alterações de sinal nos gânglios da base são vistas na RNM com ou sem acometimento de outras regiões do cérebro a fim de evitar outros diagnósticos etiológicos errôneos de patologias que cursam com demência rapidamente progressiva.

## INTRODUCTION

Subacute sclerosing panencephalitis (SSPE) is a very rare but serious complication of measles virus infection. SSPE occurs in 4-11 cases per 100,000 cases of measles. It is caused by a persistent mutant measles virus long after the acute infection.^1^ Cranial imaging studies have a limited role in the early diagnosis of the disease with descriptions of hyperintensities in posterior portions of the brain during follow-up.^2,3^

However, neuroimaging can be useful for differential diagnosis when clinical features allow characterization of a rapidly progressive dementia syndrome.^3^

The aim of this paper was to present a case of rapidly progressive dementia with unique lesions on Magnetic Resonance Imaging (MRI) in which autopsy confirmed SSPE diagnosis.

## CASE REPORT

We describe a 15-year-old boy with seizures for the last three years characterized by jerks in arms and legs as myoclonias. Early in the evolution, an improvement in seizures was observed with valproate. However, the episodes became more generalized over the last year. When returning to school after the vacation period, an unexplained decline in his school performance was noted. His parents reported that he was no longer able to write or read. A swallowing delay was also observed at the time. He had normal birth history and normal motor and mental development, with milestones at the expected age. He was correctly immunized (including for measles) with no measles history, previous epilepsy or family history of epilepsy.

On neurological examination, unmotivated laughing and intermittent myoclonus was observed. The patient exhibited grasping, snouting, paratonia mainly in upper limbs, tongue tremor and dystonia in upper and lower limbs. After two months, the patient deteriorated rapidly in motor and cognitive aspects and exhibited increased dystonia and spasticity. At this point, the dys-tonic impairment was defined as generalized and had a trunk component which rendered the patient unable to walk. Initial performance on the Mini-Mental State Examination was 10 out of 30 points.

Full blood count, urea, creatinine, electrolytes, calcium, ammonia, liver, B12 vitamin, thyroid function tests, and investigation for Wilson's disease were all negative. Rheumatological screening was negative. Antibodies for *Treponema pallidum *and HIV were also negative while IgG antibodies for measles, rubella and herpes virus were positive.

The cerebrospinal fluid (CSF) analysis, performed after 3 years of disease, showed absence of pleocytosis (2 cells). Protein concentration was slightly increased (52 mg/L), glucose level was normal (56 mg/dL) and immunoglobulin G (IgG) clearly increased (17.7 mg/L or 34.2% of total protein count-normal: 7-14%). The poly-merase chain reaction (PCR) for measles, adenovirus, cytomegalovirus, *Toxoplasma Gondii* and herpes simplex virus in CSF were negative. CSF reaction for *Treponema pallidum was negative*. CSF was assayed for measles and rubella antibodies with negative results and submitted to measles and rubella virus isolation on cell cultures from CSF samples.

**Figure 1:**
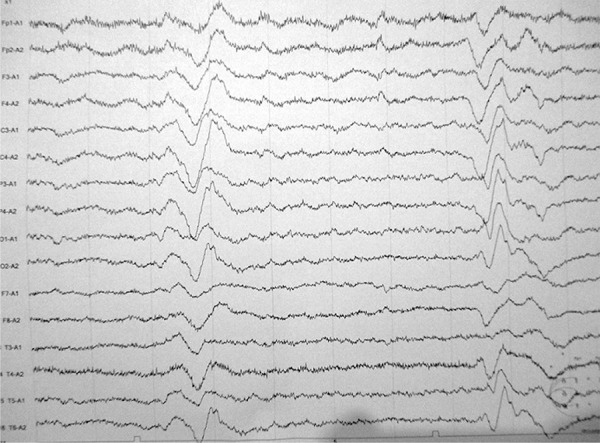
Electroencephalogram (EEG) showing generalized periodic activity at 5-6 Hz with asymmetrical background activity.

**Figure 2:**
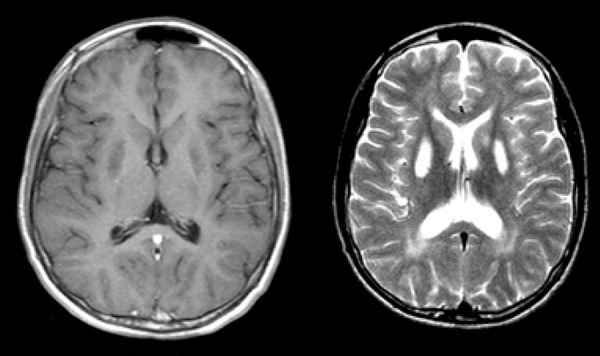
MRI showing bilateral basal ganglia lesions with hypointensity on T1- and hyperintensity on T2-weighted images.

Electroencephalogram (EEG) showed generalized periodic activity at 5-6 Hz with asymmetrical background activity, which was more disorganized in the right hemisphere, concomitant to myoclonus (Figure 1). MRI was performed and exhibited caudate and putam-inal bilateral lesions with hyperintensities on T2/Flair MRI sequences and hypointense signal on T1-weighted sequence (Figure 2). There were no hyperintensities on diffusion sequences.

**Figure 3:**
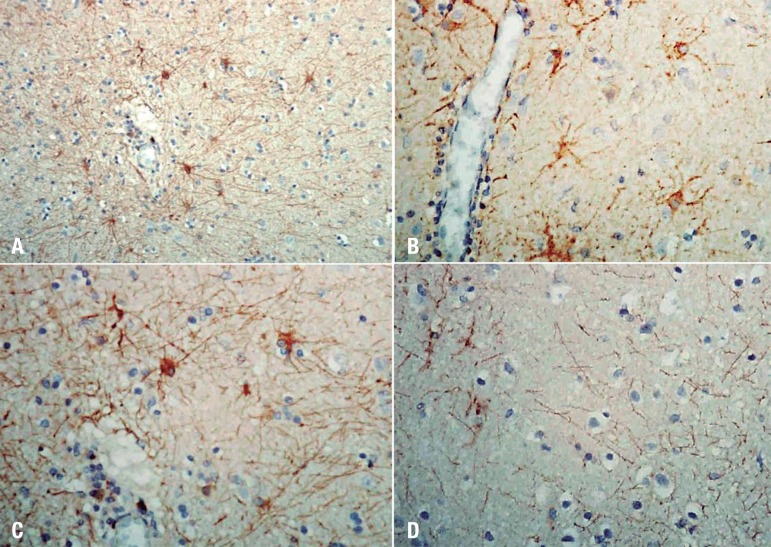
Immunohistochemistry for detection of measles antibodies in the central nervous system. [A] Multiple neurons X200. [B] Perivascular neurons displaying granular pattern of immunostaining. X400. [C] Positive immunostaining in cytoplasm and axons of neurons. X400. [D] Stained axons by specific antibody for measles. X400.

Antemortem investigations did not yield an unifying diagnosis, but after rapid evolution to severe dementia and given the EEG findings, SSPE was suspected, despite having tested negative for measles. Postmortem examination confirmed a diagnosis of subacute sclerosing panencephalitis. The neuropathological findings were perivascular inflammatory cuffing, cortical and subcortical white matter astro-microgliosis, neurono-phagia and Cowdry type "A" eosinophilic intranuclear inclusion bodies, suggesting alterations by measles. An immunohistochemistry analysis on autopsied tissue was performed and the result was positive for measles antigens (Figure 3).

## DISCUSSION

Subacute sclerosing panencephalitis is a rare progressive neurological disorder of childhood and early adolescence that is more frequent in childhood. SSPE remains an important public health issue in parts of the developing world due to limited measles immunization policies.^4,5^

Early clinical features of SSPE include myoclonus (60%), behavioral changes (13%), seizures (8%) and cognitive decline (5%). As the disease progresses, ataxia, ocular and visual manifestations (including papilloedema, retinitis, chorioretinitis, optic disk pallor, homonymous visual field deficits, and cortical blindness), pyramidal signs and dyskinesia are frequently reported^1^

Clinical diagnosis of SSPE is based on typical clinical features, periodic complexes on EEG and measles antibodies in the CSF. Criteria to reliably establish a diagnose of SSPE have been proposed and include characteristic clinical features and EEG pattern, elevated immunoglobulin levels and measles virus antibodies in the CSF together with typical histopathology on brain biopsy or postmortem examination (Table 1).^4,6^

**Table 1:** Subacute sclerosing panencephalitis (SSPE) diagnostic criteria.

**Major**	
1. Elevated cerebrospinal fluid measles antibody titres.	
2. Typical or atypical clinical history.	
	Typical: acute (rapidly) progressive; subacute progressive, chronic progressive, chronic relapsing-remitting.
	Atypical: seizures, prolonged stage I, unusual age (infancy/adult).
**Minor**	
3. Typical EEG (periodic complexes).	
4. Increased cerebrospinal fluid IgG.	
5. Brain biopsy.	
6. Special: molecular diagnostic test to identify measles virus mutated genome.	

Usually two major criteria plus one minor criterion are required; the more atypical the SSPE, the more criteria 5 and/or 6 are needed. EEG: electroencephalogram; IgG: immunoglobulin G.

There are four stages of clinical impairment described in SSPE. In Stage I, personality changes, school failure and bizarre behavior are observed. The second stage is characterized by typical periodic or quasi periodic axial myoclonic jerks whose manifestation causes recurrent falls. Generalized rigidity with extrapyramidal features and unresponsiveness appear in Stage III. Stage IV is the terminal stage of the disease and is characterized by minimal conscious state and later akinetic mutism associated with persistent high fevers and bouts of generalized sweating, both being due to autonomic failure.^4^ The prognosis of SSPE is poor with death typically occurring within 2 to 4 years of onset as no curative treatment is available.^4^ According to the rapid onset and progression of the disease, the reported patient evolved from Stage I to III within two months. Early stage changes in this case cannot be estimated as Stages I and II are usually indistinguishable.^7-9^

Common laboratory findings are elevated measles antibody titers in both CSF and serum. Mild pleocy-tosis or increased protein levels may be detected. The most remarkable feature of the CSF is the presence of a marked increase in IgG directed against the measles virus. Further investigations using isoelectric focusing can detect the presence of oligoclonal bands.^1^ Another remarkable CSF finding in this case was the gammaglobulin peak which suggests significant immune response to measles virus by the central nervous system.^5^ PCR can be negative, in chronic brain infection, not excluding diagnosis of SSPE.

Neuroimaging can be normal in up to one-third of patients. MRI findings can also change with clinical stage. The most frequently involved structures of the brain on MRI are periventricular and subcortical white matter, with parieto-occipital lobes of the cerebral hemispheres more frequently and severely affected. In later stages, progressive hemispheric, cerebellar and brain-stem atrophy occur. In the Stage IV, when the patient is in a vegetative state, almost total loss of white matter occurs and the corpus callosum also becomes thinner. At this stage there is marked cerebral atrophy. In SSPE, grey matter is less severely affected.^2,6^

At early stages, MRI shows brain edema and atrophy which remains evident during all stages of the disease. Changes in signal intensity are only evident during Stage II-III. In Stage II, the parieto-occipital white matter is predominantly affected, while diffuse fronto-pari-etal high signal intensity without contrast enhancement is common during Stages II-III.^2^ Diffusion-weighted imaging can be positive as result of membrane break-down.^6,10,11^ In a study of 76 patients with SSPE, 3 of them had basal ganglia involvement at Stage III.^12^ None of the neuroimaging abnormalities were associated with poor prognosis or clinical deterioration.^7^

This study involved a SSPE case with MRI lesions evident in bilateral basal ganglia and caudate nucleus. These findings are rare and unlike this case, when detected the cortex had already shown signs of disease. SSPE is an etiology of rapidly progressive dementia syndrome that could be suggested after application of a standardized MRI protocol which may also be useful for following disease progression.
